# Supplementary material for: Manuscript submission systems and metadata completeness in Crossref: Patterns and associations
Source: PLoS One. 2026 Mar 23;21(3):e0345417. doi: 10.1371/journal.pone.0345417 (PMC13008043; doi:10.1371/journal.pone.0345417)
Supplement: Attachment — Submitted filename: Response to reviewer.pdf [file pone.0345417.s003.pdf]

Annex

Response to Reviewers

We once again thank the reviewers for their thoughtful comments. We are happy that our revisions have been able to satisfy the reviewers. Below is a response to the remaining points that have been raised:

- Reviewer #1: The authors have covered most issues identified in the earlier review and have either explained their choices' rationale or they have adjusted the submitted manuscript.

I suggest that the authors detail (where needed) their review responses in the paper as rationale for their methodological decisions.

We have gone through our rebuttal letter and concluded that all our choices' rationale are covered in the manuscript. In two instances we found that the formulation in our rebuttal was more clear than our original formulation. We have updated the text accordingly.

- I appreciate that the several potential sources of bias or error are identified in the new "3.5 Limitations" section. Kindly verify that these include all the points raised by the reviewers.

We have gone through the reviews and can confidently say all points raised our now covered in the new limitations section.

- It would be helpful to identify the number to journals associated with publishers operating a single submission system versus the ones associated with publishers operating multiple submission systems.

We have included these on page 6
